# Barn swallows long-distance migration occurs between significantly temperature-correlated areas

**DOI:** 10.1038/s41598-018-30849-0

**Published:** 2018-08-17

**Authors:** Mattia Pancerasa, Roberto Ambrosini, Nicola Saino, Renato Casagrandi

**Affiliations:** 1 Politecnico di Milano, Dipartimento di Elettronica, Informazione e Bioingegneria, Piazza Leonardo da Vinci 32, Milano, 20161 Italy; 2 University of Milano Bicocca, Department of Earth and Environmental Sciences, Piazza della Scienza 1, Milano, 20126 Italy; 3 University of Milan, Department of Environmental Science and Policy, Via Celoria 26, Milano, 20133 Italy

## Abstract

Organisms are routinely confronted with crucial decisions on the best time and place to perform fundamental activities. However, unpredictable spatio-temporal variation in ecological factors makes life-history optimization difficult particularly for long-distance migrants, which are putatively blind of conditions thousands of kilometers and weeks ahead along their journey. Here we challenge, on a hierarchy of geographical scales, the common wisdom that migratory birds have no clue to ecological conditions at destination. Using ringing data of the inter-continental migrating barn swallow (*Hirundo rustica*), we show that temperatures at breeding sites and at times of arrival from migration are more correlated with those at actual wintering sites and at times of departure than with those at other sites and at periods before/after departure. Hence, individual swallows have clues to adjust timing of spring migration based on expected conditions at destination, and they apparently choose wintering sites to increase availability of such information.

## Introduction

In natural populations, organisms are expected to do the right thing at roughly the right place and time, under the limits set by their physiological and behavioural constraints, because natural selection strongly penalizes the individuals that do not appropriately match their activities to current environmental conditions^1,2^.

For example, plants budding and flowering at the wrong time, and animals breeding or migrating at the wrong place and time suffer severe reproduction and viability costs^3–6^. Organisms have evolved to track cyclical changes in ecological conditions, that typically occur with circannual periodicity, by exploiting environmental cues, like change in photoperiod, that announce forthcoming ecological changes^7–9^. While seasonal ecological changes occur predictably, fluctuations of major ecological factors that heavily impact on most organisms, like temperature or precipitations, over shorter time frames may occur rather unpredictably. The need to accommodate such fluctuations is the apparent evolutionary reason why many organisms have retained some level of temporal flexibility in the time schedule of their activities (‘phenology’). For example, birds at temperate latitudes adaptively time their breeding in spring according to photoperiod^7^, but they also show the ability to adjust the exact time of breeding according to the current weather conditions, that may vary unpredictably among sites and years^10,11^.

Plants and resident animals can directly sense the progress of seasonal variation in local ecological conditions and flexibly adjust their physiological state and behaviour according to environmental cues to prepare to the next shift in life-stage^12–14^. Unless sudden, abrupt changes in ecological factors occur, this affords them an opportunity of fine-tuning their life-cycle according to the current extrinsic conditions and retain (fairly) appropriate phenology. On the other hand, migratory animals that periodically move over large distances, alternating between breeding and non-breeding areas, have no direct clue as to the conditions that they will experience along their migration journey and at destination, weeks to months later. Migrants are therefore considered to be particularly susceptible to environmental uncertainty and thus, for example, to the negative effects of human-driven, rapid climate change, including advancement of spring phenology at temperate latitudes^3,15–18^ and increased frequency of extreme meteorological events^16,19–21^. The fitness advantages of appropriate timing of life-history events, however, is expected to select for the ability to capitalize on any environmental cue that allows to buffer the negative effects of environmental unpredictability on individual performance. Migratory animals may thus be expected to exploit any association that may exist between the ecological conditions during one stage of their annual life-cycle (e.g. staging in the non-breeding area, or *wintering*) with the conditions that they will later experience *en route*, during subsequent migration, or at destination^22,23^. These associations, for example, may arise because of large-scale climatic connections between distant geographical regions^24^. At one extreme, it may even be expected that, in order to appropriately tune their decisions on timing of migration and arrival to the breeding sites, migrants choose as wintering areas those where the most accurate information on future conditions at destination can be gathered. This may partly contribute to the migratory connectivity that migratory birds exhibit, whereby individuals that breed in the same geographical region also tend to share the same wintering area^25–31^, but see Finch *et al*.^32^. In addition, this may explain why migrants display some ability of tuning their arrival time according to the contingent weather conditions at destination, despite they cannot directly sense them^33,34^. However, the hypothesis that migrants have the opportunity of making decisions on their migration schedule based on expected conditions at destination and that the location of their non-breeding staging areas depends, at least partly, on the extent of information that they can gather on the ecological conditions they will experience at the other end of their migration journey, has been explored only very sparsely^24^.

Here, we use information for a uniquely large set of individuals of a long-distance migratory, insectivorous passerine bird, the barn swallow (*Hirundo rustica*), to test if correlations exist between temperatures in the wintering sites south of the Sahara Desert just before the start of northward spring migration and the temperatures at their individual breeding sites at the time of spring arrival from migration, several weeks later. We focus on temperature because this is the main driver of the progress of spring ecological events relevant to barn swallow ecology and breeding biology. Second, we test the prediction that temperatures at the sites where barn swallows spend their wintering period in Africa are highly correlated with those recorded at the breeding sites weeks later, when migration is completed, as expected under the hypothesis that individual barn swallows choose their wintering areas also depending on the level of temperature-connectivity with the breeding sites.

## Results

We relied on the information from ringing data for 270 barn swallows that were captured during breeding in Europe and were later recovered during wintering in Africa, or vice versa, during the period 1930–2009. For each individual, we estimated the putative departure time from Africa when spring migration started according to information from a recent geolocator study^35^ and from bird monitoring^36^, and spring arrival time to the breeding site in Europe according to a model of the progress of spring arrival of barn swallows^37^. For the year when each barn swallow was encountered in Europe and for each of the 29 preceding years, we computed the value of mean temperature conditions a barn swallow experienced in wintering and in breeding grounds and arranged them in two temporal vectors, named respectively as *τ*_*A*,*t*_ and *τ*_*E*,*t*_. More precisely, each of the 30 elements of vectors *τ* was calculated as the daily ground temperature averaged over a 31-days period centered on the estimated day of departure from Africa or arrival in Europe (see *Methods* for details).

### Correlation analysis at the continental scale

**Figure 1:**
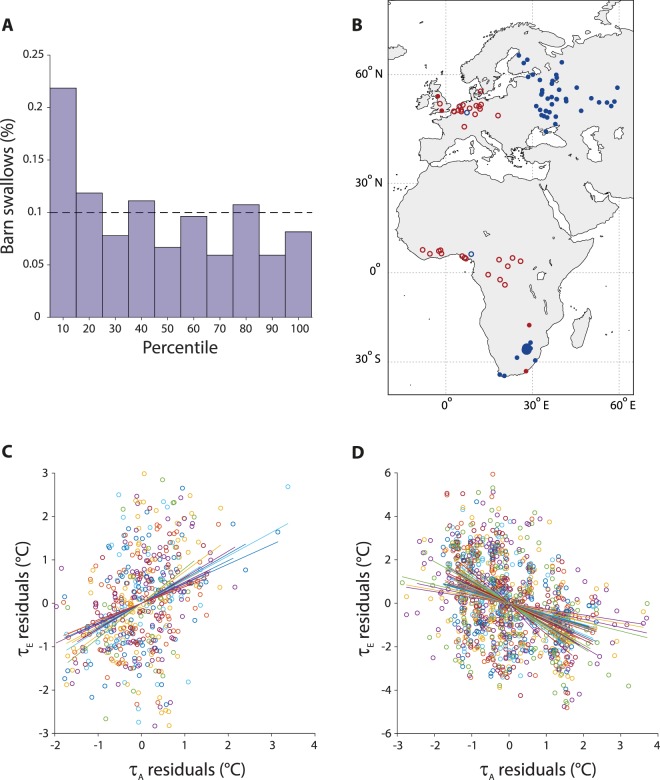
Continental scale analysis of correlations between temperatures at departure of barn swallows from Africa and at their arrival in Europe. (**A**) Ranking of partial correlations coefficients between temperatures at departure time from Africa (*τ*_*A*,*t*_, see text) and at arrival time in Europe (*τ*_*E*,*t*_). Climatic series in the actual African locations are contrasted to alternative locations in the whole sub-Saharan African subcontinent (see broad scale simulation test in *Methods*). The dashed line is the expected distribution if no site selection occurred (complete random process). (**B**) Geographical locations of the 61 barn swallows for which *τ*_*A*,*t*_ explained at least 10% of the variance of *τ*_*E*,*t*_. Circles identify individuals that winter either north of 7°S (cluster *N*) while dots those that winter south of 18°S (cluster *S*). Colors code the sign of the partial correlation coefficient between *τ*_*A*,*t*_ and *τ*_*E*,*t*_ (red for positive and blue for negative partial correlation). Dot size is proportional to the number of individuals found at any location. (**C**) Scatterplot of temperature anomalies in breeding vs wintering areas for individuals of cluster *N* whose migratory relevant temperature conditions in Africa explain more than 10% of variance of their European equivalent. Data referring to the same individual are denoted by a unique color: each circle represents the values of wintering and breeding temperatures anomalies for the focal individual in one of the 30-years of climatic reference for it. (**D**) As (**C**), but for cluster *S*.

We used partial correlation analyses controlling for temporal trends in temperature time series (*τ*_*A*,*t*_ and *τ*_*E*,*t*_) to test if the relationships with temperatures at the breeding locations in Europe were stronger for temperature time series at the actual wintering location in Africa than at other locations over the entire sub-Saharan African subcontinent. Our broad-scale simulation test (detailed in the *Methods* section) consisted in repeating 361 times the partial correlation analyses between *τ*_*E*,*t*_ and an African temperature series alternative to *τ*_*A*,*t*_. The alternative series was built exactly as *τ*_*A*,*t*_ but with temperatures evaluated in a terrestrial, non desert greographical location with coordinates taken from a regular 2° × 2° grid spanning latitudes from 15°N to 33°S and longitudes from 16°W and 50°E, i.e. covering the entire sub-Saharan African subcontinent (see Fig. S1). This analysis suggested that a large fraction of the individuals did strongly select wintering locations where the temperature correlations were stronger: indeed, for 59 (21.8%) individuals, the absolute value of the partial correlation coefficients with temperatures in their European breeding locations was higher (i.e. within the top 10%) for the actual wintering locations than for the 361 alternative locations of this broad-scale simulation test (Fig. 1A). This percentage is well above the 10% (dashed line in Fig. 1A) that would be *a priori* expected if selection did not occur.

Also, for 61 (22.6%) individuals, pre-migration temperature in the African wintering location (*τ*_*A*,*t*_) was found to explain at least 10% of the variance in temperature at the breeding site in Europe (*τ*_*E*,*t*_). The locations of these 61 individuals were clearly separated in two geographical clusters in Africa (see Fig. 1B), whose latitudes were either north of 7°S (cluster *N*) or south of 18°S (cluster *S*). Interestingly, the partial correlation coefficients between *τ*_*A*,*t*_ and *τ*_*E*,*t*_ were opposite in sign for either geographic clusters for almost all barn swallows (>95%): 18 out of 19 barn swallows whose wintering location was in Western and Central equatorial Africa (cluster *N*, Fig. 1C) exhibited positive temperature correlations (mean 0.373, standard deviation 0.056) with the relevant temperature time series at their breeding locations in Western Europe, and 40 out of 42 barn swallows wintering in Southern Africa (cluster *S*, Fig. 1D) showed negative temperature correlations (mean −0.407, standard deviation 0.064) with their breeding sites in Eastern Europe. It is noticeable that the correlations identified at departure sites were highly time-specific. Indeed, by repeating the same analysis shown in Fig. 1C,D under the hypothesis of a six weeks delayed migration, we found no significant correlations and much lower signals (see Fig. S2). Our crude clustering based on latitude of barn swallows wintering locations alone is almost identical to the one proposed by Ambrosini and coauthors^25^, which was based on migratory connectivity (see details therein). We thus found that the clustering based on geographical locations is almost indistinguishable from a specific climatic partitioning based on the signs of partial correlation coefficients between temperatures at departures and arrivals for our migratory species. These results are compatible with the idea that there might be climatic guidance to site selection for the long-distance migrating barn swallows breeding in different European regions.

### Correlations peak at the local scale

The interpretation that choice of the wintering location was guided by climatic signals is reinforced by the analysis of statistical significance of the correlation between temperatures in wintering and breeding locations at the local geographical scale. The number of individuals showing a one-tailed statistically significant correlation between *τ*_*A*,*t*_ and *τ*_*E*,*t*_ exceeded that expected by chance alone (binomial test for the deviation of the proportion of significant correlations from 0.05: *p* < 0.00001 in both clusters). Because spatial autocorrelation of temperature may violate the binomial test assumption of independence of the observations, we ran a local-scale randomization test. At each of 999 replications, we randomly extracted one position within 1000 km of the wintering site of each individual and calculated the partial correlation between temperatures at the random African position (${\tau }_{{{\rm{A}}}_{{\rm{rand}}},{\rm{t}}}$) and temperatures at the breeding site (*τ*_*E*,*t*_) (see *Methods* for details). The number of significant correlations between *τ*_*A*,*t*_ and *τ*_*E*,*t*_ (18 for cluster *N* and 42 for cluster *S*) was larger than the number of correlations between ${\tau }_{{{\rm{A}}}_{{\rm{rand}}},{\rm{t}}}$ and *τ*_*E*,*t*_ for all the 999 replications (number of significant correlations from randomization: cluster *N*: mean ± SD: 7.28 ± 2.08, range: 1–13; cluster *S*: 27.45 ± 3.79, range 17–39). Hence, the temperature correlations with the breeding location appeared to be consistently stronger for the actual wintering location than for other random locations within the same African region.

**Figure 2:**
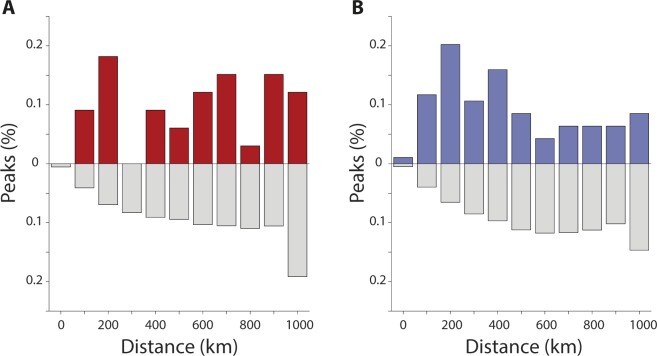
Frequency distributions of the distances of the correlation peak between temperatures relevant to migration from Africa to Europe and (red or blue bars) the actual wintering locations of barn swallows, or (grey bars) the randomly generated positions in Africa (local scale randomization, see *Methods*) in either cluster *N* (**A**, red) or cluster *S* (**B**, blue).

To further investigate the spatial pattern of variation of the strength of the correlation within the region surrounding the actual wintering sites, we first calculated the correlations between temperatures at the nodes of a grid 1000 km in radius and centered on the wintering site (see green symbols in Fig. S1) and temperatures in the breeding site (*τ*_*E*,*t*_). Then, we searched for the node where the largest significant correlation occurred and named it ‘correlation peak’. The frequency distribution of the distances from the grid center (i.e. the wintering site) where such correlation peaks occurred showed that a large proportion of them (27.27% for cluster *N*; 32.29% for cluster *S*) was located within 200 km of the wintering site (red bars in Fig. 2), a distance that is of the same order of magnitude of the individual home range of barn swallows during wintering in Africa (see *Methods*). To test if the frequency distribution of correlation peaks resulted from spatial autocorrelation of temperatures (i.e. from a pure climatic process), rather than from site selection, we re-ran the same analysis 999 times by centering the grids at each run on a random position in Africa within 1000 km from actual location as in the local scale randomization described in *Methods*. In both clusters *N* and *S*, the observed frequency distributions markedly differed from those obtained by randomizations (Fig. 2). Indeed, the frequency distribution for the actual wintering locations (red or blue bars in Fig. 2) were hump-shaped, with a mode rather close to the actual wintering position (*ca*. 200 km), whereas the frequency distribution from the randomized locations (grey bars) increased with distance, with local maxima around 800 km in cluster *N* (Fig. 2A) or 600 km in cluster *S* (Fig. 2B).

To compare these patterns, we analyzed the results of the 999 local-scale randomizations of Fig. 2 and counted the number of times where the fraction of correlation peaks occurring within 200 km was higher at the actual locations of barn swallows (red or blue bars) than at random ones (grey bars). The result was unequivocal: this occurred 992 (99.2%) times in cluster *N* and always (999 times; 100%) in cluster *S*. If the correlation peaks had been randomly distributed around the center of our grid, the probability of finding correlation peaks within a distance of 200 km would have been around 4% (i.e. the ratio between the areas of two circles with radius 200 and 1000 km, respectively). The fact that the frequency distributions of correlation peaks obtained for the actual wintering sites markedly differed from those obtained for the locally randomized positions in both clusters strongly supports the idea that the two processes have different nature and that, also at a local geographical scale, barn swallows choose as wintering locations those where temperatures have stronger correlation with temperatures in the breeding locations.

### Robustness

**Figure 3:**
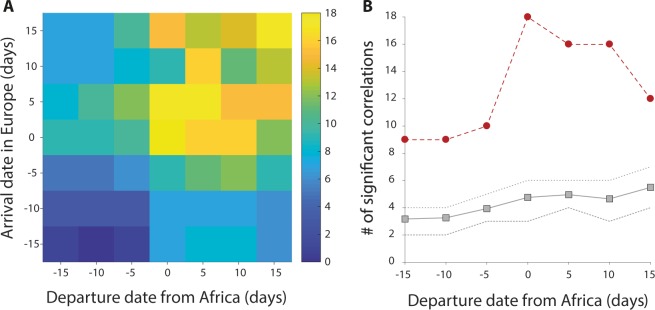
Temporal sensitivity analysis. (**A**) The number of significant positive partial correlations for barn swallows in cluster *N* at different temporal shifts (positive meaning delay) of the departure date from Africa (*x*-axis) and the arrival date in Europe (*y*-axis). (**B**) Sensitivity of significant correlations between *τ*_*A*,*t*_ and *τ*_*E*,*t*_ to temporal shifts of the departure date using actual positions in Africa (red circles) or 999 local randomized positions (gray squares, see *Methods*). Dotted gray lines indicate the 25–75 percentiles.

We tested for robustness of these patterns of correlation with respect to the choice of the reference time periods assumed for departure from Africa and arrival to Europe by shifting these periods by a maximum of 15 days in both directions (back and forth), in steps of five days. A maximum of significant correlations clearly emerged for the time periods that we used in the analyses above, especially for cluster *N* (see Fig. 3A). We remark that such periods considered to be relevant for migration were chosen purely on the basis of the information currently available on the actual phenology of barn swallow migration (see Introduction). The most important axis of variation in our temporal sensitivity analysis is the shifting of departure date from Africa, because it is known with less accuracy than time of arrival to Europe. While holding the time of arrival to Europe constant (time shift on the *y*−axis = 0 in Fig. 3A), but shifting the departure time from Africa, the number of significant correlations between *τ*_*A*,*t*_ and *τ*_*E*,*t*_ in the actual wintering sites markedly outnumbered that obtained from 999 local scale randomizations of positions (see Fig. 3B). Interestingly, the maximum number of significant correlations was achieved exactly for the chosen departure period (i.e. for temporal shift in Africa equal to 0). The result obtained in cluster *S* was weaker and showed that there might be a slight increase of partial correlations with increasing shifts of the period of departure from Africa (see Fig. S3).

## Discussion

Every spring, approximately 2.1 billion songbirds and near-passerine birds belonging to 68 species leave their wintering areas in Africa and head to their breeding quarters in Europe^38^. The timing of spring migration is believed to be deeply rooted in endogenous, genetic mechanisms^8,39,40^. However, considerable annual variation exists in the time of spring migration and arrival to the breeding quarters within individual breeding populations, and such temporal variation in migration phenology has cascading effects on the timing of breeding and thus on population productivity^41,42^. Variation in spring phenological events is remarkably associated with meteorological conditions at the destination of the migration journey, with lower temperatures and, for example, later timing of snow melting at the breeding grounds being associated with delayed arrival^11^. Inter-annual adjustment of spring arrival dates requires a mechanism that allows migrants to tune migration phenology according to the conditions ahead. It has been speculated that migrants adjust the progress of migration along their journey^43–45^, although fueling during migration stopovers may not be advantageous for several migratory species. Tuning of the pace of migration while migrants are *en route* may not be the sole mechanism causing apparently adaptive variation of arrival time depending on conditions at destination. Using a single long-distance migratory species for which considerable information on wintering areas exist, here we speculated that cues may exist that allow individuals to adjust their timing of departure from the wintering site, not only the pace at which they proceed along their migration journey. This requires that correlations exist between the ecological conditions at departure and conditions at destination around the expected time of arrival, several weeks later and thousands of kilometers ahead^24^. The first implication of the present analyses is that such for our reference species *Hirundo rustica* correlations do indeed exist, suggesting that migratory barn swallows may in fact obtain some information on the temperatures at destination while they are still on their wintering grounds.

### Tuning migration schedules

Appropriately shifting the time of arrival depending on the conditions at destination can have major positive effects on individual performance. Early arrival affords a number of social advantages including access to the best breeding territories, as well as advantages in the mate choice process and in sperm competition^46–48^. On the other hand, too early arrival relative to the current ecological conditions as determined for example by temperature, which is the main driver of spring phenological events at temperate and boreal latitudes, can entail major fitness costs in terms of survival or physiological state via an effect on food availability^21,39^. Information on future temperatures at destination may thus allow migratory barn swallows to optimize the resolution of the trade-off between the benefits and costs of arriving early.

The time-lagged temperature correlations that we documented were not generally very strong, as could be expected. However, mild advantages arising from tuning migration schedules according to even inaccurate information on future conditions at destination can provide a selective advantage when confronted with the alternative strategy of turning down any chance of exploiting existing cues.

### Wintering sites selection

We also provided an indirect test of the actual effect of temperature correlations between the wintering and the breeding sites on migration and wintering strategies. If such correlations are influential on life-history decisions, it could be expected that the sites that individual birds choose to spend their non-breeding season are non-randomly located with respect to their temperature correlations with the breeding sites. In fact, our second major finding was that the wintering sites of individual barn swallows in sub-Saharan Africa have larger temperature correlations with the breeding site than with other locations in Africa. This pattern held both at the subcontinental scale of sub-Saharan Africa and at a local geographical scale (i.e. within 1000 km from the actual wintering location). This might indeed suggest that the choice of the wintering location partly functions to maximize the information on annual conditions at the breeding site that is available before the start of migration.

### Climate and migratory connectivities

Notably, the sign of the temperature correlations between the wintering and the breeding sites varied between barn swallow geographical populations that could be identified based on an analyses of migratory connectivity^25^. South-Western European barn swallows experience positive correlations between temperatures in wintering and breeding sites and are considerably more likely to winter in equatorial Africa, whereas North-Eastern European barn swallows experienced negative correlations and are mostly found wintering in southern Africa. The ecological and evolutionary mechanisms that cause migratory connectivity of animal populations are largely unknown and probably consist of a blend of geographical and historical effects. Our study suggests that differential climatic correlations between European breeding areas and the African wintering regions that are connected to them by migration of barn swallows may have a role in the evolution of migratory connectivity of barn swallows. If temperature correlations between the wintering and breeding sites drive the choice of wintering location, as our analyses at two geographical scales suggest, it may be inferred that climatic correlations between continents select for migratory connectivity because of the advantages of exploiting the information on future conditions at the breeding sites that is provided, for individual European regions, by temperatures in different regions of sub-Saharan Africa.

Temperature correlations between the wintering and the breeding sites may also contribute to the mechanism by which migratory birds are responding to climate change effects. Several trans-Saharan migratory species are advancing the time of spring arrival to the breeding sites in Europe and this has been attributed to the advantages of keeping track of rapid advances in spring phenological events that occur in Europe. If current climate change is preserving the existing patterns of spatio-temporal correlations between temperatures in sub-Saharan Africa and in Europe, we may expect that these correlations will boost the process of adjustment of migration phenology to current climate change. On the other hand, disruption of temperature correlations will hinder adjustment adaptive adjustment of migration phenology with negative effects on populations of long-distance migrants.

Our results lead to hypothesize that other migratory species of birds and other taxa may also use information from environmental cues at one end of their migration journey or *en route* to adaptively modulate their migration phenology. This hypothesis may be tested using existing historical information from ringing data or by relying on rapidly accumulating information from recently-developed migration tracking devices. In addition, they suggest that the existence of time-lagged climatic correlations can have a role in shaping the evolution of migration strategies and in the choice of the wintering sites. Whether this will boost the ability of populations to cope with the effects of current climate change or, conversely, hinder their ability to provide adaptive response to climate change will largely depend on whether differential climate in the breeding and in the non-breeding staging areas disrupts the existing climatic correlations between areas. The long-distance migratory species of the Afro-Palearctic system show broad variation in the area encompassed by both their breeding and wintering ranges (see http://www.iucnredlist.org/), although most of these species have both large wintering and breeding ranges. The present results on the barn swallow are therefore particularly relevant to the interpretation of the effects of large-scale climatic correlations on the expression and evolution of migration behaviour and migratory connectivity of widely distributed species, for which differential climatic connections between parts of the wintering and breeding ranges can occur.

## Methods

### Ringing data

Ring recoveries were obtained from the EURING^49^ and SAFRING^50^ databanks. Out of the 73610 records in the original datasets, we selected the 270 individuals ringed and re-encountered (i.e. recaptured alive or found dead, in any circumstance, ‘ring re-encounters’ hereafter) during stationary periods in the breeding and the wintering grounds. In particular, those periods are December-February in Africa (south of the Sahara Desert) and June-July in Europe and North Africa (see the study of Ambrosini and coauthors^25^ for a similar approach). Selected ring re-encounters spanned 1930–2009 and were within 34.46°N–65.07 °N and 4.53°W–59.67°E in the breeding grounds and within 7.75°N–34.45 °S and 7.92°W–35.00°E in the wintering grounds.

Ringing data show high spatial and temporal heterogeneity^51^, however in this work we retained all data because the relevant information for the present study is the geographical location of the breeding and the wintering sites of the single individuals, which is clearly well represented by its ringing and recovery locations.

### Departure and arrival dates of spring migration

We first estimated the arrival dates from spring migration in each breeding location according to Ambrosini and coauthors^37^, which reports a map of estimated arrival dates of the first 15% of barn swallows in Europe and North Africa (10°W–26°E, 26°N–66°N, resolution 4° × 4°, latitude × longitude) based on an analysis of ringing data spanning 1908–2008. We estimated the arrival date at each breeding location by ordinary kriging with a stable variogram model (sensu Wackernagel^52^) of estimated arrival dates at each cell as identified in Ambrosini and coauthors^37^. The kriging algorithm was used also to extrapolate the arrival dates of barn swallows ringed or recovered in Europe outside the area covered by that study^37^ (i.e. to the east of 26°E, *n* = 88 individuals). However, to reduce uncertainty in the estimated arrival dates for these latter individuals, we used their median estimated arrival date (7 May = i.e. Julian date 125) as the arrival date of all the individuals breeding to the east of 26°E.

At present, we are aware of no model allowing an estimation of departure dates from each African location. For all barn swallows wintering south of 7°S (see Results), we used the mean departure date from South Africa as reported in Altwegg and coauthors^36^ (10 February = 41). For all barn swallows wintering north of 7°S (see Results), we selected the mean departure dates reported in Liechti and coauthors^35^ (2 March = 61).

### Large-scale, long-term temperature data

To test for correlations between temperatures in the wintering and breeding locations of each individual in the focal period of spring migration, we used temperature data obtained from the atmospheric reanalysis ERA20C of the European Centre for Medium-Range Weather Forecasts^53^. The ERA20C dataset was chosen for two reasons. First, because instrumentally recorded temperature data are not available with adequate resolution (neither in space nor in time) for the whole study areas, particularly in Africa. Second, because among the different global reanalyses currently available, ERA20C is the one with the finest spatial horizontal resolution (approximately 125 km) for the period 1900–2010. Operatively, we started from the 2-metre temperature data at 6 hours intervals over a 1° × 1° geographical grid and calculated mean daily temperatures at each point. Then, we computed mean daily temperatures at the two exact coordinates where each barn swallow was ringed or recovered by spatial bilinear interpolation. In this manner, we could obtain a time series of mean daily temperatures from 1900 to 2010 for the exact geographical positions where each barn swallow was ringed and re-encountered. From each time series, as detailed below, we extracted a short-term time series *τ*_*E*,*t*_ [*τ*_*A*,*t*_] by selecting temperatures that are relevant to spring migration in Europe [Africa] in the 30-years interval ending with the year of recovery in Europe. We opted for a 30-years timespan in analyzing temperatures, in accordance with suggestions by the World Meteorological Organization, because we wanted to work over temporal windows which are relevant to climatic rather than meteorological conditions. In order to smooth daily fluctuations and obtain temperature conditions of year *t* which are relevant to influence departure of barn swallows from Africa (*θ*_*A*,*t*_) and their arrival to Europe (*θ*_*E*,*t*_), we averaged all temperatures within the 31 days centered, respectively, on the specific departure and arrival dates described above. Thus, two migratory-relevant temperature series, computed over a climatic suitable and consistent temporal horizon, were assigned to each individual: one for the African location, *τ*_*A,t*_ = [*θ*_*A,t*_, *θ*_*A,t*−1_, …, *θ*_*A,t*−29_] and one for the European location *τ*_*E,t*_ = [*θ*_*E,t*_, *θ*_*E,t*−1_, …, *θ*_*E*,−29_].

### Continental-scale randomizations

In order to test for climatic correlations between breeding and wintering locations of individual barn swallows, we first calculated the partial correlation coefficient (*r*_par_) between *τ*_*A*,*t*_ and *τ*_*E*,*t*_ at African and European actual locations, i.e. the locations retrieved from the ringing data, while controlling for the effect of year. To assess whether such partial correlation coefficients were significant at a broad (continental) spatial scale, we then computed the same partial correlation coefficients for all individuals by holding the European location and year fixed and using 361 additional positions in Africa. The African additional positions used for this broad spatial scale simulation were distributed according to a 2° × 2° regular grid (south of 15 °N) whose nodes did not occur neither on the sea nor in desert areas (see exemplificative cyan dots in Fig. S1). Desert locations were identified as those where the average daily Total Precipitation (as retrieved from ERA20C reanalyses) was lower than 0.65 mm over the period 1900–2010^54^. The absolute values of the 362 partial correlation coefficients (one between the actual wintering and breeding locations plus 361 for the simulations over the entire sub-Saharan Africa) calculated for each barn swallow were then listed in decreasing order of magnitude, thus obtaining the rank of the value for the correlation between the actual wintering and breeding positions.

### Local-scale randomizations

A second type of spatial randomization test was then performed to check the robustness of our results at a finer (‘local’) geographical scale. To this end, we assessed whether the number of significant partial correlations was larger than those arising from a null model where the positions of barn swallows in Africa were randomly selected 999 times within a distance of 1000 km around the true wintering position, while avoiding points located on the sea or in desert areas (see for example the portion of the yellow circle in Fig. S1).

It is worth noticing that there is an important asymmetry in the relationships between recorded locations of barn swallows captured (or found) in Europe and in Africa. While the home range radius of individuals in the European breeding grounds is of about 1 kilometer^55^, in the African wintering areas it can be as large as hundreds of kilometers^56^. It is therefore important to explore whether there are any structured spatial patterns of partial correlations surrounding the actual location where the barn swallow has been found in Africa, e.g. the location is close to a *correlation peak*. To investigate this possibility, we computed the partial correlation coefficients between *τ*_*A*,*t*_ and *τ*_*E*,*t*_ also for a set of points that were homogeneously distributed in the region surrounding the wintering position. To this end, we built a radial grid of points (filled green squares in Fig. S1) centered on each of the barn swallow locations. Nodes of every grid were placed at 45° to one another (starting from the north), at distances of 100–1000 km (at 100 km intervals) from the central position (green dot in Fig. S1). Grid nodes located on the sea or in desert areas (see above) were disregarded (open squares in Fig. S1). For each barn swallow, at each grid node we calculated the partial correlation between the local temperatures and those at the European breeding location and recorded the distance from the grid center of the node where correlation was significant and strongest (the *correlation peak*).

### Temporal sensitivity analysis

Finally, since there is some uncertainty on the exact period of migration of each individual, we also analyzed the sensitivity of the number of significant correlations to changes in the dates of both departure from Africa and arrival to Europe. This was done by advancing or delaying of at most 15 days (with steps of 5 days) the dates of departure and/or arrival over which *τ*_*A*,*t*_ and *τ*_*E*,*t*_ were computed.

## Electronic supplementary material

Supplementary Information
