# Supplementary material for: Barn swallows long-distance migration occurs between significantly temperature-correlated areas
Source: Sci Rep. 2018 Aug 17;8:12359. doi: 10.1038/s41598-018-30849-0 (PMC6098147; doi:10.1038/s41598-018-30849-0)
Supplement: Supplementary file 1 — Supplementary Information [file 41598_2018_30849_MOESM1_ESM.pdf]

Supplementary Information
Barn swallows long-distance migration occurs between significantly temperature-correlated areas

Mattia Pancerasa[1], Roberto Ambrosini[2,*], Nicola Saino[3,*], and Renato Casagrandi[1,*]

[1]Politecnico di Milano, Dipartimento di Elettronica, Informazione e Bioingegneria, Piazza Leonardo da Vinci 32, Milano, 20161, Italy
[2]University of Milano Bicocca, Department of Earth and Environmental Sciences, Piazza della Scienza 1, Milano, 20126, Italy
[3]University of Milan, Department of Environmental Science and Policy, Via Celoria 26, Milano, 20133, Italy
[*]roberto.ambrosini@unimib.it, nicola.saino@unimi.it, renato.casagrandi@polimi.it

ABSTRACT

This Supplementary Information includes three supplementary figures aimed at (S1) representing the randomization tests defined in the Methods section of the main article, (S2) contrasting temperatures anomalies at breeding vs wintering positions (as in Fig. 1 of the main article) under the assumption of a migration period delayed by six weeks, and (S3) performing for cluster *S* the same analysis shown for cluster *N* in Fig. 3 of the main article.

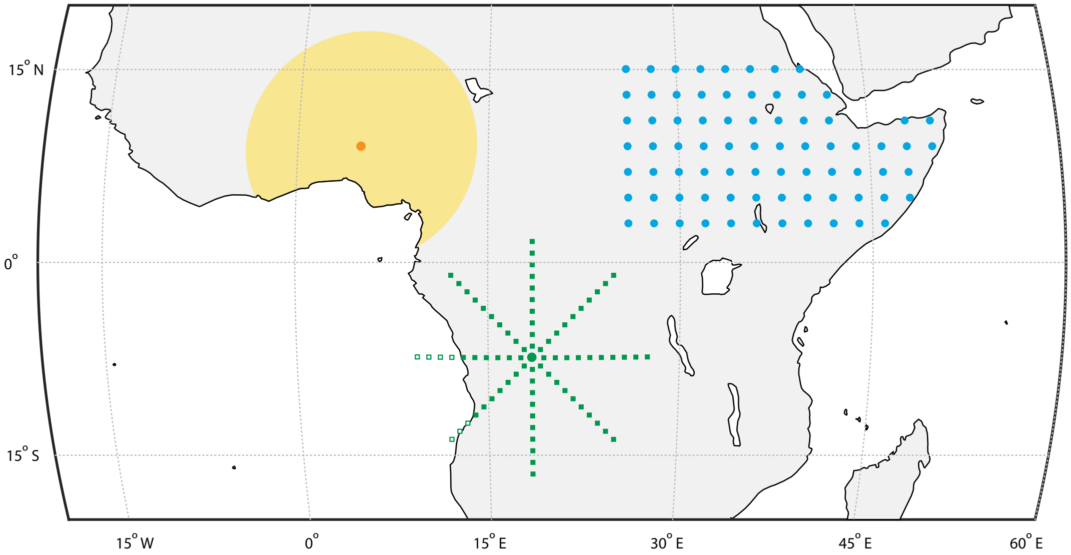

Figure S1. Exemplificative representation of randomization tests used to evaluate the strength of the correlations between the African and the European temperatures relevant to barn swallow migration and their correlation peaks. Cyan dots are some of the 361 nodes of the sub-Saharan Africa subcontinent grid (2°×2°) used to perform the broad scale simulation test. The circular yellow area (radius of 1000 km) is where we randomly extracted (with a uniform probability distribution) the locations alternative to the focal barn swallow wintering location (orange center) to perform the local scale randomization test (ocean and desert areas are excluded). The wind rose-like radial grid surrounding a hypothetical barn swallow wintering location (green) used in our simulation to search for correlation peaks: filled green squares are for locations where r_{par} between $\tau_{A,t}$ and $\tau_{E,t}$ was computed, white filled squares were discarded locations. See the Methods section in the main text for further details.

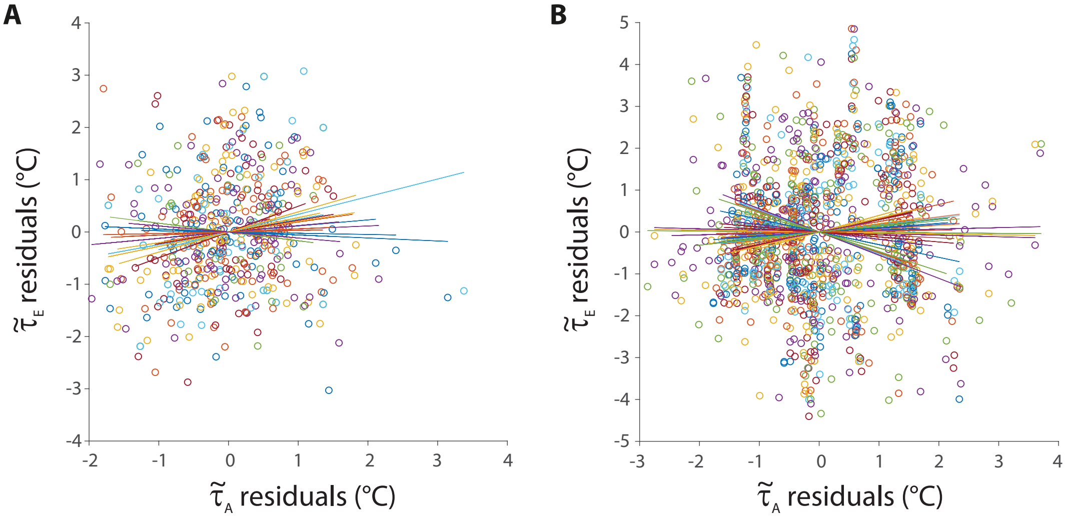

Figure S2. Scatterplots of temperature anomalies in breeding vs wintering areas for individuals of (**A**) cluster *N* and (**B**) cluster *S* computed as in Fig. 1 in the main text, but for temperature series in Africa ($\tilde{\tau}_A$) and Europe ($\tilde{\tau}_E$) evaluated six weeks after the estimated migration time.

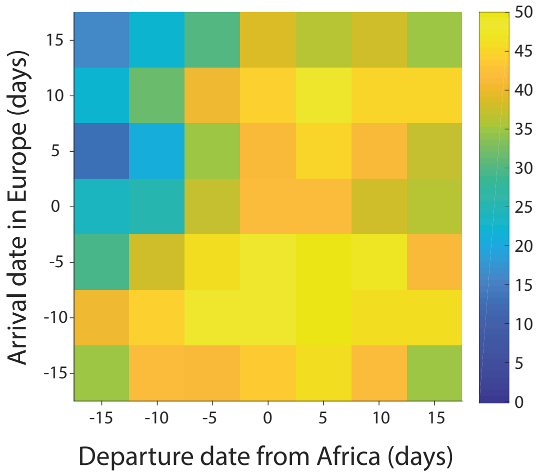

Figure S3. Number of significant negative partial correlations for barn swallows in cluster *S* for different temporal shifts (positive meaning delay) of the departure date from Africa (*x*-axis) and the arrival date in Europe (*y*-axis). See main text and Fig. 3A therein for further information.
